# Selection and Use of Online Learning Resources by First-Year Medical Students: Cross-Sectional Study

**DOI:** 10.2196/mededu.7382

**Published:** 2017-10-02

**Authors:** Terry Judd, Kristine Elliott

**Affiliations:** ^1^ Department of Medical Education University of Melbourne Parkville Australia; ^2^ Centre for the Study of Higher Education University of Melbourne Parkville Australia

**Keywords:** information-seeking behaviors, educational technologies, e-learning, learning analytics

## Abstract

**Background:**

Medical students have access to a wide range of learning resources, many of which have been specifically developed for or identified and recommended to them by curriculum developers or teaching staff. There is an expectation that students will access and use these resources to support their self-directed learning. However, medical educators lack detailed and reliable data about which of these resources students use to support their learning and how this use relates to key learning events or activities.

**Objective:**

The purpose of this study was to comprehensively document first-year medical student selection and use of online learning resources to support their bioscience learning within a case-based curriculum and assess these data in relation to our expectations of student learning resource requirements and use.

**Methods:**

Study data were drawn from 2 sources: a survey of student learning resource selection and use (2013 cohort; n=326) and access logs from the medical school learning platform (2012 cohort; n=337). The paper-based survey, which was distributed to all first-year students, was designed to assess the frequency and types of online learning resources accessed by students and included items about their perceptions of the usefulness, quality, and reliability of various resource types and sources. Of 237 surveys returned, 118 complete responses were analyzed (36.2% response rate). Usage logs from the learning platform for an entire semester were processed to provide estimates of first-year student resource use on an individual and cohort-wide basis according to method of access, resource type, and learning event.

**Results:**

According to the survey data, students accessed learning resources via the learning platform several times per week on average, slightly more often than they did for resources from other online sources. Google and Wikipedia were the most frequently used nonuniversity sites, while scholarly information sites (eg, online journals and scholarly databases) were accessed relatively infrequently. Students were more likely to select learning resources based on the recommendation of peers than of teaching staff. The overwhelming majority of the approximately 70,000 resources accessed by students via the learning platform were lecture notes, with each accessed an average of 167 times. By comparison, recommended journal articles and (online) textbook chapters were accessed only 49 and 31 times, respectively. The number and type of learning resources accessed by students through the learning platform was highly variable, with a cluster analysis revealing that a quarter of students accessed very few resources in this way.

**Conclusions:**

Medical students have easy access to a wide range of quality learning resources, and while some make good use of the learning resources recommended to them, many ignore most and access the remaining ones infrequently. Learning analytics can provide useful measures of student resource access through university learning platforms but fails to account for resources accessed via external online sources or sharing of resources using social media.

## Introduction

### Background

Medical students routinely turn and are directed toward online information sources and services [1-2]. Course-related learning activities are typically well-supported with resources delivered through institutional learning platforms, while university libraries provide access to online collections of reliable and authoritative biomedical science and clinical resources. The ready accessibility of these resources drives educator expectations that students will locate, access, and assimilate them to support their learning. However, medicine is one of the most time-poor and information-rich professions, and when faced with too much information, too many choices, and not enough time, students may resort to superficial information seeking and retrieval strategies [3-4].

The first year of the medical curriculum often emphasizes biosciences over clinical learning. Students draw on a range of information to support their bioscience learning, although lectures and lecture materials continue to play a central role. Knowledge gained through lectures is often assimilated or applied to the clinical context through problem- or case-based learning approaches. One of the key assumptions underpinning these approaches is that students will self-direct their learning. That is, they will independently locate, access, and assimilate appropriate information to build their knowledge base and develop the necessary understanding and experience to apply and transfer this knowledge. Previous generations of medical students would have relied heavily on their own lecture notes, key textbooks, and physical access to their library. This scenario has clearly changed over the past 20 or more years as online resources have grown in number, quality, and availability. While data on the decline in student use of their own notes and textbooks is scant and largely anecdotal, the decline in physical use of academic libraries is clearly documented. Martell [5], for example, describes changing usage patterns among the 124 North American Association of Research Libraries (ARL) libraries between 1995 and 2006, documenting an overall decline in circulation of 26%—and 58% for specialist medical libraries—over that time. More recent data from ARL libraries [6] and from our own university library suggest that this trend is continuing, with a 70% fall in print circulation in our own biomedical library between 2006 and 2015. However, the number, variety, and academic use of electronic library resources has increased dramatically over the same period. Again, reliable usage data are somewhat hard to come by although Martell [5] reports an approximately 440% increase in electronic transactions (to approximately 5.7 million per year) for Harvard University’s library from 2001 to 2006. Our own university experienced a more than doubling of accesses of key eBook collections (to around 1.5 million) between 2013 and 2015. While the number and scope of scholarly articles or documents available to students will vary somewhat from institution to institution, recent estimates put the number of documents indexed by Google Scholar at around 160 million [7].

Despite students having ready access to such a broad range of scholarly resources (eg, online journals and databases), including many high-quality biomedical science and clinical resources, previous studies suggest that many medical students tend to rely on a limited number of resources and resource types [8-9]. While not specifically referring to medical students, Head [10] talks about student use of “tried and true” resources, which typically include course readings, Google, Wikipedia, and—less frequently—library databases. Not all of these resources necessarily meet expected standards, leading to concerns that medical student information-seeking strategies may favor convenience and expediency over quality and reliability [2,11-13].

Against such concerns, current students are also more likely to be explicitly provided with or at least directed to key learning resources. Detailed lecture notes and lecture recordings are routinely provided through institutional learning platforms, and the provision of links to recommended texts, websites, and scholarly articles means that access is usually only a click away. Increasingly, there is an expectation on the part of students that these resources will be provided to them. This feeds an expectation by teaching staff that the resources will be used by students, ensuring that key curriculum content is delivered and that student self-directed learning activities are supported. It also helps to justify the additional demands and costs associated with the production and delivery of quality online learning materials. Yet, despite the widespread adoption of online learning platforms, most of which can produce detailed information about their access and use (ie, learning analytics), we still know relatively little about the number, type, and sources of learning resources that medical students routinely select and access. Moreover, learning analytics can only provide part of the picture as they often only capture resource access and use that occurs through institutional learning platforms, ignoring student use of favored sites and tools like Google, Wikipedia, and, increasingly, social media for sharing information and resources [2,14].

The primary aim of this study then was, where practical, to document and analyze medical students’ selection and use of (primarily online) learning resources. This should help us to identify which types of learning resources are most used and most useful, and conversely, those that are underused or less useful. In addition, we sought to assess whether student selection and use of the resources aligned with medical educator and information specialist expectations. That is, to what extent does the identification, provision, and recommendation (either explicit or implied) of learning resources to students drive their use. Such findings should be of considerable interest to medical educators and scholarly information specialists (librarians), whose responsibility it is to identify, develop, and deliver effective learning resources to support medical students’ learning.

### Study Context

The purpose of this section is to provide context for the measurement, analysis, and interpretation of resource selection and use by first-year medical students. While it describes key elements of a specific medical curriculum, most of these elements, and the learning and teaching approaches they embody, are not unique.

The Melbourne Doctor of Medicine (MD) is a full-time, masters-level course. The first year takes place on campus and delivers biomedical science-oriented lectures and practicals within a framework of small group tutorials focused around weekly clinical cases. A supplementary series of small group tutorials is designed to prepare students for the clinical phase of the course (years 2 to 4).

The first-year curriculum is designed to consolidate student biomedical science knowledge and prepare them for clinical placements. Most learning activities and content are embedded within 2 year-long subjects. Foundations of Biomedical Science (FBS) comprises a mix of biomedical science lectures, clinical cases, and practical classes and is designed to develop and consolidate student knowledge across the main bioscience and biomedical disciplines. Concepts and content are taught using an integrated body systems approach with an emphasis on the application of bioscience knowledge in a clinical context. Principles of Clinical Practice (PCP) introduces and develops a series of core clinical skills, including the medical interview, physical examination, and diagnostic reasoning. Delivered through a series of weekly small group tutorials, PCP topics and activities are aligned with the body systems framework of FBS and are designed to emphasize the links between student biomedical science knowledge and clinical practice.

Clinical cases within FBS are delivered using a case-supported learning (CSL) approach. CSL encourages hypothetical reasoning and is designed to help students construct mechanistic representations of normal and abnormal processes based on their developing bioscience knowledge. CSL is delivered via small group tutorials at the beginning and end of each week. Tutors introduce the case during the first tutorial and assist students to identify salient learning issues and how these might be investigated. Students carry out these investigations during the week through a combination of self-directed and collaborative learning, which takes place around a program of lectures and tutorials. Students share their findings during the second tutorial and with the tutor’s assistance develop a comprehensive pathophysiological mechanism to explain the case.

Delivery of the first-year curriculum is supported by MD Connect, a bespoke learning platform developed within our medical school, that has over 3000 users comprising students, teaching staff, clinicians, and administrative support staff. It provides full curriculum mapping and timetabling within which learning events such as lectures, tutorials, and practicals are linked to curriculum resources. First-year students interact with the learning platform via a series of activity- and resource-based interfaces including the following:

- Timetable: a personalized weekly timetable/calendar with embedded links to activity-based learning resources
- Curriculum: a navigable curriculum map with embedded links to activity-based learning resources
- Search: simple searching of curriculum resources
- Library: a curated selection of open and subscription-based scholarly resources

### Online Resources

The learning platform mediates access to a comprehensive set of high-quality bioscience, biomedical, and clinical resources. These are drawn from an extensive curriculum database plus a selection of scholarly information sources and repositories. The curriculum database contains a detailed map of the formal curriculum with extensive linking between learning activities and supporting resources. All resources are tagged based on a series of contextual criteria. Key resource types are outlined in Table A of Multimedia Appendix 1.

Students access learning resources through the learning platform in a variety of ways. Curriculum resources (ie, resources that are explicitly mapped to formal learning activities) are typically accessed via the Timetable or Curriculum interfaces. Timetabled activities provide links to any associated learning resources, allowing for direct access. The Curriculum interface provides a navigable map of the curriculum down to the level of individual learning activities. Resources are linked to these activities as in the Timetable interface. The Library interface aggregates key bioscience, biomedical, and clinical resources and services, including academic journals, online textbooks, scholarly databases (eg, PubMed, Web of Knowledge), and clinical resources (eg, Clinical Key, BMJ Best Practice). Individual library resources are also linked to specific learning events within the curriculum database and can be accessed via the Timetable and Curriculum interfaces.

## Methods

The study draws on 2 main sources of data: a detailed survey of MD student selection and use of learning resources and learning analytics based on log file analysis of student use of the learning platform.

### Resource Use Survey

A paper-based survey of MD student selection and use of (primarily online) learning resources was administered to all first-year students in October 2013. Permission to administer the survey was granted by the human ethics committee of our university, and participation in the survey was optional and anonymous. The full survey contained 28 items (most of which contained a series of subitems) organized into 5 distinct sections covering student demographics, resource and information seeking, resource sharing, resource types, and the timing of resource use. Only data relating to items from the resource and information seeking section are relevant to this study and presented here. The items in that section were primarily designed to assess the frequency with which students access learning resources through the learning platform and from other sources. However, they also queried student perceptions of the usefulness, quality, and reliability of these resources; their sources; and their motivation for selecting particular resources or resource types. All items required participants to respond by selecting an option on a 5-point Likert scale. Frequency of use items were scored according to: 1=less than monthly, 2=less than weekly, 3=once or twice a week, 4=on most days, and 5=more than once per day. Usefulness items were scored from 1=not at all useful to 5=extremely useful. The quality and reliability items were scored from 1=very low to 5=very high, and agreement items were scored from 1=strongly disagree to 5=strongly agree. A total of 72.7% (237/326) of first-year students returned survey responses of which 36.2% (118/326) were complete for the resource and information seeking section. Only those 118 survey responses were analyzed for this study.

### Learning Platform Analytics

Detailed logs of first-year student use of the learning platform were captured over an entire semester (July to December 2012; 325 of 337 enrolled students used the learning platform during this period). Logs were captured on a per user per session basis and consisted of a detailed sequence of user actions or data requests (events), with each event described by a type, context, and timestamp. It is important to note that the survey and the log data are drawn from successive first-year student cohorts rather than the same cohort as, due to technical changes in the learning platform, detailed usage data were not available for 2013.

### Data Analysis

The survey data were analyzed using a combination of descriptive and comparative statistics and exploratory cluster analysis. Likert responses were interpreted as interval rather than ordinal data [15] allowing comparisons of related groups of items to be carried out using 1-way repeated measures analyses of variance. Comparisons of individual items within these groups were conducted using pairwise *t* tests applying the Bonferroni correction to reduce the likelihood of type I errors. Variation between individual responses was explored using k-means cluster analysis. Determination of an appropriate number of clusters was informed by plotting the percentage variation in the within-groups sum of squares values for a range of k (where k equals the number of clusters) values and identifying the k value beyond which further reduction in the within-group sum of squares was reduced [16]. All analyses were carried out using the R Studio software package (The R Foundation).

Analysis of the learning platform log data was also descriptive and exploratory. Raw log data was processed, abstracted, and analyzed using custom parsing routines to produce a series of simple measures of resource use based on which students accessed them, how they were accessed (ie, which interface within the learning platform was used to access them), and the type of learning activity they were associated with.

Variation in access patterns between users was again explored through k-means cluster analysis. The data matrix for this analysis consisted of a binary access value for every learning resource accessed by at least 1 first-year student during the target semester.

## Results

### Resource Use Survey

The results of the survey items are presented in Tables 1-3. These 3 tables contain abbreviated descriptions of the items rather than the actual wording of the item. The values are means of the item responses (based on a 5-point Likert scale), and in each case the 1-way repeated measures analyses of variance conducted on these groups of items revealed highly significant differences between them (*P*<.001).

Students reported accessing resources through the learning platform several times a week on average, slightly more frequently than they did for other online sources. Only 11 of the 118 students reported accessing learning resources from the learning platform less often than weekly, and only 2 reported that the resources they accessed through it were not useful. Physical textbooks were used less frequently (approximately weekly). When using the learning platform, students reported they were more likely to access resources through the Timetable interface (approximately daily) than through the Curriculum, Search, or Library interfaces (once or twice a week). When finding and accessing online resources from outside the learning platform, students most often turned to general search engines and Wikipedia (approximately daily) and Facebook (several times per week). The university library and Google Scholar were used less often (approximately weekly). Students also regularly sought advice on their learning from their peers—up to several times a week versus less than weekly from teaching staff.

In line with the responses to the frequency of use items, students reported the learning platform as being more useful than other online sources for locating and accessing learning resources (Table 2). The learning platform’s Timetable interface was particularly highly rated (useful to extremely useful). When seeking resources from outside the learning platform, students rated general search engines and Wikipedia as being slightly less useful than the learning platform’s Timetable interface but significantly more useful than either Facebook, Google Scholar, or the university library (Table 2). In terms of the quality and reliability of learning resources located and accessed via the various sources, students again rated the learning platform most highly (Table 3). This was followed by other online sources generally, the learning platform’s Library interface, and general search engines. Wikipedia was rated the lowest for both quality and reliability.

**Table 1:** Means of responses (5-point Likert scale to indicate less than monthly to more than daily) for survey items relating to the frequency of use of different learning resources or information sources.

Survey item		Mean^a^
**Frequency of locating/accessing resources using...**		
	Learning platform generally	3.9 BC
	Other online sources generally	3.5 C
	Physical textbooks	2.8 D
	Learning platform’s Timetable interface	4.5 A
	Learning platform’s Curriculum interface	3.4 C
	Learning platform’s Search interface	2.9 D
	Learning platform’s Library interface	2.9 D
	General search engines (including Google)	4.5 AB
	Wikipedia	4.3 AB
	Facebook	3.6 C
	University library	2.9 DE
	Google Scholar	2.6 EF
**Frequency of seeking advice from...**		
	Peers	3.7 C
	Teaching staff	2.0 F

^a^Means with nonoverlapping letter codes are significantly different (*P*<.05).

**Table 2:** Means of responses (5-point Likert scale to indicate not at all useful to extremely useful) for survey items relating to the usefulness of learning resources or information sources.

Survey item		Mean^a^
**Usefulness for finding/accessing resources of...**		
	Learning platform generally	4.0 B
	Other online sources generally	3.7 C
	Learning platform’s Timetable interface	4.4 A
	Learning platform’s Library interface	3.4 B
	General search engines	4.3 BC
	Wikipedia	4.2 BC
	Facebook	3.0 DE
	University library	3.3 D
	Google Scholar	2.9 E

^a^Means with nonoverlapping letter codes are significantly different (*P*<.05).

**Table 3:** Means of responses (5-point Likert scale to indicate very low to very high) for survey items relating to quality and reliability of learning resources from different sources.

survey item		Mean^a^	
		Quality	Reliability
**Resources available through...**			
	Learning platform generally	4.2 A	4.0 AB
	Learning platform’s Library interface	3.7 BCD	3.8 BCD
	Other online sources generally	3.9 AB	3.7 CD
	General search engines	3.7 BCD	3.6 CD
	Wikipedia	3.6 CD	3.3 D

^a^Means with nonoverlapping letter codes are significantly different (*P*<.05).

### Cluster Analysis

Inspection of the within-group sum of squares data suggested a 4-cluster solution. Membership and descriptions of these 4 clusters (groups) were as follows, with the text in parentheses indicating the approximate frequency of use or level of usefulness, quality, or reliability of the mentioned type of resource or method of accessing it.

Membership of all 4 groups was characterized by regular access of resources through the learning platform (on most days) and attribution of considerable value (useful to extremely useful) to the learning platform for locating resources and to the resources accessed through it. The median frequency of access of online resources from sources other than the learning platform varied between once or twice a week (groups 2 and 4) and on most days (groups 1 and 3).

Members of group 1 (n=22) were less likely than those in all other groups to agree that their selection of learning resources was influenced by external factors (eg, available time, convenience, recommendation by others). They were also most likely to access learning resources through the university library (on most days). Membership of group 2 (n=29) was characterized by less frequent searching for resources using either Google (on most days; all other groups reported using it more than daily) or the learning platform’s Search function (less than weekly). They were also much less likely to use or find Facebook useful for accessing learning resources (less than weekly and not at all useful). Group 3 members (n=35) were the most likely to use Google, Wikipedia, and Facebook for accessing learning resources (more than daily). They were also more likely to find these sites useful and to rate the quality and reliability of resources they accessed through these sites highly. Members of group 4 (n=32) were the least likely to use physical textbooks or seek advice from teaching staff (less than monthly). They were also much less likely to access learning resources through the university library, either directly, through the learning platform, or via Google Scholar (less than weekly) or to find these sources of information useful.

### Learning Platform Analytics

A total of 44,222 discrete sessions on the learning platform (per user median 118, maximum 577) with a median cumulative session time of 73.9 hours were logged and analyzed. That equates to an average of 4.5 sessions and 2.8 hours per user per week. Of the almost 1 million individual user actions or events that were captured, just over half were associated with use of the platform’s Timetable interface. Users accessed 71,101 curriculum resources during the target semester, with most (90.99%) being accessed through the platform’s Timetable interface.

### Resource Use by Learning Platform Interface, Event, and Resource Type

Table B of Multimedia Appendix 1 lists the types and number of timetabled learning events and the number of resources linked to each through the Timetable and Curriculum interfaces. Table C provides a breakdown of the number and use of resources linked to timetabled learning events by resource type (see Table A also).

A total of 264 unique learning activities and 1079 linked resources were timetabled during the target semester. The most common timetabled learning activities were lecture (170/264), CSL tutorial (36/264), practical (24/264), and PCP tutorial (18/264). Almost two-thirds of all linked resources (685/1079, 63.48%) were directly associated with or derived from lectures (eg, lecture notes, lecture videos, or audio recordings), with the next most common resource types being journal articles (87/1079, 8.06%), websites (51/1079, 4.73%), and CSL case notes (36/1079, 3.36%).

Of those resources linked directly to lectures, 95.0% (685/721) were either lecture notes or lecture recordings. Lecture notes attracted the highest level of use, with each set being downloaded an average of 168.8 times and each user downloading an average of 93.7 different lecture note resources. Downloads of other resource types linked to lectures ranged from extremely low (eg, lecture audio: 2.9 downloads per resource) to moderately high (eg, textbook: 140 downloads per resource).

A further 358 resources were linked to timetabled events other than lectures, including CSL tutorials, PCP tutorials, and practicals. The more common resource types linked to these events included journal articles (72/358, 20.1%), websites (51/358, 14.2%), PCP roleplays (36/358, 10.1%), case notes (36/358, 10.1%) and textbooks (29/358, 8.1%).

**Table 4:** Proportions of users accessing resources via the learning platform’s Timetable interface.

Resource type	Activity type	Resources	% Usage I^a^	% Usage II^b^
All	All	1079	100	98.2
Lecture notes	FBS^c^ lecture	187	95.4	50.6
Lecture video^d^	FBS lecture	166	74.2	10.4
Journal article	FBS lecture	15	23.1	6.5
	CSL tutorial	69	71.1	15.1
CSL case notes	CSL tutorial	36	48.3	7.6
CSL video	CSL tutorial	16	42.5	6.9
Website	CSL tutorial	46	56.6	7.1
	FBS tutorial	4	11.1	5.8
Image	CSL tutorial	15	58.5	17.2
Textbook	CSL tutorial	29	52.0	7.9
PCP video	PCP tutorial	30	50.8	15.3
PCP roleplay	PCP tutorial	36	37.2	16.5
Tutorial notes	FBS tutorial	6	52.9	15.2
Reading	FBS practical	10	79.7	35.4
	FBS tutorial	9	70.8	48.9
Extras	FBS lecture	17	75.1	25.4
	CSL tutorial	3	14.5	6.7
	FBS practical	9	51.4	10.8

^a^Percentage of students who accessed at least 1 resource.

^b^Average number of those resources accessed per student as a percentage of the number of resources.

^c^FBS: Foundations of Biomedical Science.

^d^Downloadable videos only.

Access of resources via the learning platform’s other interfaces was comparatively low and variable. For example, of the 34 resources available via the Library interface, 32 were accessed by at least 1 user. However, of these, only 7 of 23 recommended textbooks and 1 of 4 resource collections (MD Consult) were accessed on more than 50 occasions.

### Resource Access by User

Table 4 details the proportion of users accessing different types of learning resources through the learning platform’s Timetable interface by learning activity.

All users viewed at least 1 timetabled activity and virtually all (319/325, 98.2%) accessed at least 1 resource via the learning platform’s Timetable interface. Lecture notes were accessed by almost all users (310/325, 95.4%), with each accessing just over one-half (94.3/187, 50.4%) of the available lecture notes resources. Almost three-quarters of users (241/325, 74.2%) also accessed lecture videos but they did so much more selectively, accessing only 10.4% (17.3/166) of the available recordings on average (Table 4). Readings also attracted comparatively high levels of access, with a clear majority of users viewing at least 1 reading associated with an FBS tutorial (230/325, 70.8%) or practical (259/325, 79.7%). Access rates for most other resource types and activity combinations were much more selective. Less than one-quarter of users (75/325, 23.1%) accessed any journal articles that were linked to lectures and, on average, each user accessed only 1 of these resources. Users were much more likely (231/325, 71.1%) to access journal articles associated with CSL tutorials, however.

Access rates for resources via other interfaces were low by comparison. Around half (159/325, 48.9%) of users accessed at least 1 resource by the Search or Curriculum interfaces and by the Library interface (183/325, 56.3%), and two-thirds (216/325, 66.5%) accessed at least 1 software resource. However, only around 1 in 3 users (119/325, 36.6%) accessed 10 or more resources via an interface other than the Timetable.

### Cluster Analysis

Examination of the within-group sum of squares data suggested a 5-cluster solution. Membership and characteristics of the 5 clusters (groups) are described below according to the following usage level categories, where usage level refers to the proportion of members accessing a particular type of learning resource: very low≤5%, low=6% to 10%, moderately low=11% to 20%, moderate=21% to 40%, moderately high=41% to 60%, high=61% to 80%, and very high=81% to 100%.

Group 1 (n=42) was characterized by high use of lecture notes, moderately high use of downloadable lecture videos, moderately low use of journal articles, and low to moderately low use of case notes, textbooks, and websites. Group 2 (n=91) was characterized by moderately high use of lecture notes and low to very low use of all other resource types. Group 3 (n=25) was characterized by very high use of lecture notes and low to very low use of other resource types. Group 4 (n=95) was characterized by very high use of lecture notes, moderately low to moderately high use of journal articles, and low to moderate use of lecture videos, CSL case notes, textbooks, and websites. Group 5 (n=84) was characterized by low use of lecture notes and very low use of all other resource types.

## Discussion

### Principal Findings

Despite clear areas of overlap, the survey and analytics data paint somewhat different pictures of student selection and use of resources. Analysis of the survey data suggests a pattern of regular access (on most days) by students of resources via the learning platform and from other online sources only slightly less frequently. Google and Wikipedia were also frequently used as sources of information or as starting points for locating information. This is despite students rating them significantly lower than the learning platform for quality and reliability, which is consistent with recent studies of first-year medical student information-seeking behavior [11,13]. Access of scholarly information sources, whether via the learning platform, university library, or Google Scholar, was typically infrequent.

The analytics data, on the other hand, reveal a pattern of variable access and use of resources via the learning platform, with most use concentrated around specific resource types and users. Lecture notes and readings aside, many learning resources only appear to be used by a small percentage of users (see Table 4). In addition, the cluster analysis of the analytics data reveals a surprisingly large subset of users (group 5) who accessed very few resources, including lecture notes, via the learning platform. This group accounted for approximately 25% of students within the 2012 first-year MD cohort. The cluster analysis of the survey data provides a different perspective again. There is no clear low usage group in this case; the approximately 9.3% of respondents (11/118) who reported accessing resources through the learning platform least often (less than weekly) being spread throughout the 4 groups, with groups differentiated largely on the basis of lower or higher use of or preference for particular resource types or access methods (eg, low use of textbooks by group 4; more frequent use of Google, Wikipedia, and Facebook by group 3).

If we accept that the analytics data are reliable and representative of student information-seeking behavior, then for those students who accessed the recommended resources infrequently (group 5 in the cluster analysis of the analytics data), a possible consequence of their behavior is that they are less well prepared and informed than their peers. This could impact their academic performance. Evidence linking or even comparing general resource use and academic performance appears to be limited, however. Goodall and Pattern [17] suggest the existence of positive relationships between library use and academic performance among undergraduate students at their university but failed to test these relationships statistically. Huon and colleagues [18] describe weak but significant correlations between resource use and academic performance among a group of first-year psychology students for some resource types—textbooks (*r*^2^=0.21) and discussion forums (*r*^2^=0.15)—but found no relationship for other common resources including lecture notes or tutorial materials. Further investigation of such relationships, and for medical students specifically, seems warranted given the limited and equivocal nature of these studies.

With respect to the majority of students who regularly used learning resources, their strong reliance on lecture notes confirms previous findings that these continue to form a key part of student learning strategies [10]. While students appeared to rate lecture notes highly (based on their assessments of the usefulness, quality, and reliability of resources accessed via the learning platform, see Tables 2 and 3), they are neither designed nor intended to meet all of our medical students’ expected learning needs. Huon and colleagues [18] argue that student resource selection and use is driven much more by assessment needs than by exploring for understanding. Despite the learning and teaching approaches underpinning our and many other medical curricula (ie, case-based and self-directed learning), this is likely true for our students as well. If a deep knowledge and understanding of the curriculum is reflected in the breadth and depth of learning resources investigated, then based on the data presented here perhaps only a minority of students (best represented by group 4 in the cluster analysis of the analytics data) might be well placed to achieve this.

### Limitations

While each of the general findings described above are potentially important and likely to have wider implications, there are some clear limitations to the study that need to be acknowledged. These include our focus on a single curriculum and the implementation of a specific (and specialized) learning platform, although both would appear to be representative of other medical curricula and sorts of learning platforms they employ. As with similar surveys, there are questions of accuracy and reliability of the questionnaire data, given the reliance on student perceptions and recollections of past resource use. The analytics data, on the other hand, has a high level of accuracy but is limited in its scope. It reliably captures when, what, and how students access resources from within the learning environment but reveals little about their discovery and use of other online learning resources.

Student use of social media is a case in point here. Just under a quarter of students who responded to the survey reported using Facebook on an approximately daily basis for accessing learning resources. In a related study [14], more than half of the surveyed medical students reported using Facebook and other technologies (primarily email and cloud-based file storage and sharing services) to share learning resources with their peers on most days. This includes resources that were originally sourced through the learning platform, which could in part explain the low usage rates of some learning resources (eg, lecture video recordings) in this study. The importance of these sharing networks in medical student learning practices is poorly understood, probably underestimated, and warrants further investigation.

### Conclusion

As previously mentioned, the relationships between resource discovery and use, learning, and academic performance are yet to be properly explored. Assessing these relationships in a way that accurately and reliably captures typical student study practices and controls for past performance appears challenging but could provide valuable insights into medical student learning behavior and the effectiveness of various types of resources to inform and support their learning.

## Appendix

Multimedia Appendix 1 Resource types and usage.

## Abbreviations

ARL: Association of Research Libraries
CSL: case-supported learning
FBS: Foundations of Biomedical Science
MD: Melbourne Doctor of Medicine
PCP: Principles of Clinical Practice
