# Supplementary material for: Selection and Use of Online Learning Resources by First-Year Medical Students: Cross-Sectional Study
Source: JMIR Med Educ. 2017 Oct 2;3(2):e17. doi: 10.2196/mededu.7382 (PMC5643842; doi:10.2196/mededu.7382)
Supplement: Multimedia Appendix 1 [file mededu_v3i2e17_app1.pdf]

A. Curriculum resource types associated with timetabled learning activities.

**Type**	**Description**
Lecture notes	PDF notes created from lecture presentations (PowerPoint)
Lecture recording	Video and/or audio capture files of lecture presentations including lecture slides and lecturer audio
Journal article	Full text journal articles
Textbook	Electronic versions of key bioscience and biomedical textbooks
Website	Consumer, practitioner and researcher oriented websites
Image	Image files (often used to support CSL cases)
CSL case notes	PDF notes containing details of CSL cases (separate files for first and second tutorials)
CSL video	Video clips designed to contextualize CSL cases
PCP roleplay	PDF notes designed to support PCP roleplay exercises
PCP video	Video clips designed to support PCP learning activities (includes videos of physical examination and medical interview techniques)
Tutorial notes	PDF notes containing details of non-CSL FBS tutorials
Reading	PDF or web notes designed to be read before specified lecture or tutorial activities
Extras	Collection of minor resource types

B. Number of timetabled learning events and linked resources for the main types of events.

**Event type**	**Events**	**Resources**
All	264	1079
Lecture	170	721
CSL tutorial	36	227
PCP tutorial	18	66
Practical	24	29
Tutorial (other)	6	22
Clinical colloquium	6	14
Other	4	0

C. Number and use of resources linked to timetabled learning activities within the learning platform. Usage calculated as the total number of access requests divided by the number of resources.

**Resource type**		**Resources**	**Use**
All		1079	53.8
Lecture notes		191	166.9
Lecture video	(download)	169	34.7
	(streaming)	169	9.4
Lecture audio		169	2.8
Journal article		87	48.8
Textbook		30	30.7
Website		51	23.3
Image		18	78.4
CSL case notes		36	25.5
CSL video		16	23.4
PCP roleplay		36	55.7
PCP video		30	51.5
Tutorial notes		6	51.2
Readings		21	130.4
Extras		38	13.4
